# Supplementary material for: Tumor Lymphocyte Infiltration Is Correlated with a Favorable Tumor Regression Grade after Neoadjuvant Treatment for Esophageal Adenocarcinoma
Source: J Pers Med. 2022 Apr 13;12(4):627. doi: 10.3390/jpm12040627 (PMC9029859; doi:10.3390/jpm12040627)
Supplement: Supplementary file 1 [file jpm-12-00627-s001.zip › jpm-1483921-supplementary.pdf]

Table S1. (Supplementary): The density of comparison markers in benign versus malignant stroma and tumors.

TIL	Stroma Malignant	Stroma Adjacent	P value	Tumor Malignant	Tumor Adjacent	P value
CD3	21.2±16.1	21.8±16.6	0.97	8.3±9.6	6.7±10.3	0.41
CD20	4.6±5.4	3.6±4.1	0.29	1.3±2.5	1.1±2.0	0.7
CD8	15.1±15.7	12.3±11.3	0.3	11.3±17.0	5.4±7.6	0.03
CD4	8.7±8.1	12.1±8.2	0.034	4.9±6.6	3.0±4.6	0.13
CD45RO	13.4±10.3	15.8±7.6	0.16	4.3±4.7	6.0±5.6	0.09
FOXP3	2.8±3.3	3.0±3.4	0.76	2.2±2.0	2.0±3.1	0.69
CD68	20.4±15.0	17.9±8.7	0.39	7.4±4.7	6.2±10.8	0.59
CD163	22.7±19.5	20.8±6.7	0.57	6.2±5.8	5.7±10.6	0.84

TIL, tumor infiltrating lymphocytes.

Table S2. (Supplementary): Quantification of tumor infiltrating lymphocytes in the tumor microenvironment in the different patient groups.

	All Cohort						
TIL	(N=43)<br><br>% post cell (Mean±SD)	% post cell (Median)	Chemo only (N=28) % patients low vs. high	Surgery only<br><br>(N=15) % post cell (Mean±SD)	Good Responder (N=13) % post cells (Mean±SD)	Bad Responder (N=15) % post cells (Mean±SD)	P value
CD3-Stroma	17.5±15.1	13	45 : 55	18.2±18.4	30.2±10.5	9.1±4.9	0.003
CD3-Tumor	6.3±7.9	2	9 : 91	4.3±6.7	15.3±8.8	2.5±1.6	0.001
CD20-Stroma	3.4±3.9	2	50 : 50	3.9±4.7	3.9±3.4	2.5±3.4	0.63
CD20-Tumor	1.1±2.1	1	46 : 54	0.9±0.7	1.2±0.7	1.2±2.9	0.91
CD8-Stroma	11.1±11.1	8.5	30 : 70	12.1±15.5	16.6±3.6	7.1±6.7	0.13
CD8-Tumor	6.4±10.1	2.0	44 : 56	2.6±2.4	20.6±14.8	2.6±2.7	0.001
CD4-Stroma	8.7±7.4	6.5	52 : 48	7.9±5.9	15.3±6.3	6.1±7.7	0.013
CD4-Tumor	3.0±4.3	1.5	48 : 52	2.1±3.8	6.6±6.1	1.8±2.2	0.004
CD45R0-Stroma	11.9±8.2	10	39 : 61	11.6±6.6	16.9±7.5	9.5±9.2	0.11
CD45R0-Tumor	3.8±4.2	2	31 : 69	1.8±1.4	8.5±4.7	3.4±4.2	0.001
Foxp3-Stroma	2.4±2.7	1	42 : 58	2.1±1.6	3.9±4.7	2.0±2.2	0.3
Foxp3-Tumor	1.7±2	1	30 : 70	1.2±1.5	3.4±3.0	1.4±1.2	0.03
CD68-Stroma	16.0±9.9	14	41 : 59	15.2±8.2	15.6±15.6	17.3±7.1	0.85
CD68-Tumor	5.9±5.8	4	41 : 59	4.8±2.5	4.8±3.3	7.9±9.0	0.33
CD163-Stroma	18.0±11.6	15	36 : 64	17.8±12.5	18.6±13.4	17.8±9.9	0.99
CD163-Tumor	4.7±5.5	3	27 : 73	3.4±3.6	5.4±4.7	5.7±7.6	0.51

TIL, tumor infiltrating lymphocytes; Post, positive.

Table S3. Quantification of tumor infiltrating lymphocytes in the tumor microenvironment of chemotherapy treated groups.

	Area	Good Responders (N=13)	Bad Responders (N=15)	P Value
CD3	Stroma	30.2±10.5	9.1±4.9	0.001
	Tumor	15.3±8.8	2.5±1.6	0.001
CD20	Stroma	3.9±3.4	2.5±3.4	0.37
	Tumor	1.2±0.66	1.2±2.8	0.99
CD8	Stroma	16.6±3.6	7.1±6.7	0.001
	Tumor	20.6±14.8	2.6±2.8	0.001
CD4	Stroma	15.3±6.3	6.1±7.7	0.009
	Tumor	7.3±5.8	1.8±2.2	0.004
CD45R0	Stroma	16.9±7.5	9.5±9.2	0.066
	Tumor	8.5±4.7	3.4±4.2	0.014
FOXP3	Stroma	3.9±4.7	2.0±2.2	0.24
	Tumor	3.4±3.0	1.4±1.2	0.04
CD68	Stroma	15.6±15.6	17.3±7.1	0.72
	Tumor	4.8±3.3	7.9±9.0	0.34
CD163	Stroma	18.6±13.4	17.8±9.9	0.87
	Tumor	5.4±4.7	5.7±7.6	0.93

Table S4. (Supplementary): Quantification of tumor infiltrating lymphocytes by lymph node involvement / staging in chemotherapy treated groups.

	**Area**	**LN-N0 (N=13)**	**LN-N+ (N=15)**	**P value**	**Stage I (N=10)**	**Stage II (N=3)**	**Stage III (N=15)**	**P value**
CD3	Stroma	28.5±11.5	8.8±4.9	0.001	33.1±9.4	17.7±7.2	8.8±4.9	0.001
	Tumor	14.5±8.7	2.1±0.8	0.001	16.7±9.6	9.3±2.5	2.1±0.8	0.001
CD8	Stroma	15.9±4.0	6.9±6.9	0.002	16.6±3.9	13.5±4.9	6.9±6.9	0.008
	Tumor	19.0±14.7	2.4±2.7	0.001	18.9±15.0	19.5±19.1	2.4±2.7	0.002
CD4	Stroma	17.0±7.9	4.4±3.6	0.001	14.7±6.7	25±8.45	4.4±3.6	0.001
	Tumor	7.0±5.5	1.6±2.1	0.003	7.4±6.2	5.5±0.7	1.6±2.1	0.01
CD45R0	Stroma	19.2±9.9	7.4±4.9	0.001	16.7±8.1	28.0±14.1	7.4±5.0	0.001
	Tumor	9.2±4.9	2.6±2.8	0.001	8.4±5.0	12±4.2	2.6±2.8	0.001

LN-N0: lymph node without metastases, LN-N1 – lymph node with metastases.

Table S5. The relationship between tumor infiltrating lymphocyte subpopulations enrichment and disease free and overall survival.

	DFS			OS		
	Low Enrichment Mean (95%CI) months	High Enrichment Mean (95%CI) months	P value	Low Enrichment Mean (95%CI) months	High Enrichment Mean (95%CI) months	P value
CD3 Stroma	9.5 (5.3:13.7)	58.0 (33.3:82.8)	0.001	19.6 (15.1:24.0)	59.0 (39.0:79.0)	0.001
CD3 Tumor	5.0 (0:12.8)	38.1 (20.2:55.9)	0.074	14.5 (7.6:21.4)	43.8 (29.1:58.4)	0.021
CD8 Stroma	18.9 (14.2:23.6)	59.7 (39.1:80.3)	0.001	20.3 (16.2:24.5)	67.2 (42.5:91.9)	0.001
CD8 Tumor	9.3 (5.0:13.7)	43.3 (21.5:65.0)	0.077	189 (15.5:22.2)	54.3 (35.0:73.5)	0.002
CD4 Stroma	13.9 (2.6:25.2)	51.5 (25.8:77.2)	0.047	23.6 (13.9:33.4)	60.5 (39.1:81.8)	0.005
CD4 Tumor	15.7 (4.3:27.0)	47.0 (21.0:73.0)	0.096	28.0 (16.4:36.3)	53.4 (31.8:75.0)	0.11
CD45R0 Stroma	16.9 (2.1:31.6)	41.0 (18.6:63.4)	0.35	27.4 (15.5:39.3)	50.4 (30.8:70.1)	0.12
CD45R0 Tumor	8.3 (3.5:13.1)	43.7 (22.1:65.3)	0.021	17.7 (12.7:22.7)	52.8 (33.3:72.3)	0.003

DFS, disease free survival; OS, overall survival.
